# Increased CSF-decorin predicts brain pathological changes driven by Alzheimer’s Aβ amyloidosis

**DOI:** 10.1186/s40478-022-01398-5

**Published:** 2022-07-04

**Authors:** Richeng Jiang, Una Smailovic, Hazal Haytural, Betty M. Tijms, Hao Li, Robert Mihai Haret, Ganna Shevchenko, Gefei Chen, Axel Abelein, Johan Gobom, Susanne Frykman, Misaki Sekiguchi, Ryo Fujioka, Naoto Watamura, Hiroki Sasaguri, Sofie Nyström, Per Hammarström, Takaomi C. Saido, Vesna Jelic, Stina Syvänen, Henrik Zetterberg, Bengt Winblad, Jonas Bergquist, Pieter Jelle Visser, Per Nilsson

**Affiliations:** 1 Department of Neurobiology, Care Sciences and Society, Center for Alzheimer Research, Division of Neurogeriatrics, Karolinska Institutet, 171 64 Stockholm, Sweden; 2 Department of Otolaryngology Head and Neck Surgery, The First Hospital of Jilin University, Changchun, 130021 China; 3 Department of Neurobiology, Care Sciences and Society, Center for Alzheimer Research, Division of Clinical Geriatrics, Karolinska Institutet, 141 52 Huddinge, Sweden; 4 Department of Clinical Neurophysiology, Karolinska University Hospital, Stockholm, Sweden; 5 Alzheimer Center Amsterdam, Department of Neurology, Amsterdam Neuroscience, Vrije Universiteit Amsterdam, Amsterdam UMC, 1007 MB Amsterdam, The Netherlands; 6 Department of Neurosurgery, The Second Affiliated Hospital of Shaanxi Chinese Medicine University, Xianyang, 712000 Shaanxi China; 7 Division of Physiology and Neuroscience, Carol Davila University of Medicine and Pharmacy, 050474 Bucharest, Romania; 8 Department of Chemistry – BMC, Analytical Chemistry and Neurochemistry, Uppsala University, 752 37 Uppsala, Sweden; 9 Department of Biosciences and Nutrition, Karolinska Institutet, 141 52 Huddinge, Sweden; 10 Department of Psychiatry and Neurochemistry, Institute of Neuroscience and Physiology, The Sahlgrenska Academy at the University of Gothenburg, 413 45 Mölndal, Sweden; 11 Clinical Neurochemistry Laboratory, Sahlgrenska University Hospital, 413 45 Mölndal, Sweden; 12 Laboratory for Proteolytic Neuroscience, RIKEN Center for Brain Science, Wako, Saitama 351-0198 Japan; 13 IFM-Department of Physics, Chemistry and Biology, Linköping University, 581 83 Linköping, Sweden; 14 Molecular Geriatrics, Department of Public Health and Caring Sciences, Uppsala University, 751 85 Uppsala, Sweden; 15 Department of Neurodegenerative Disease, UCL Institute of Neurology, London, WC1N 3BG UK; 16 UK Dementia Research Institute at UCL, London, WC1E 6BT UK; 17 Hong Kong Center for Neurodegenerative Diseases, Hong Kong, China; 18 Theme Inflammation and Aging, Karolinska University Hospital, 141 52 Huddinge, Sweden; 19 Alzheimer Center Limburg, School for Mental Health and Neuroscience, Maastricht University, 6211 LK Maastricht, The Netherlands

**Keywords:** Alzheimer’s disease, Decorin, Cerebrospinal fluid, Mass spectrometry, *App* knock-in mice, Amyloid-β (Aβ), Choroid plexus, Extracellular matrix, Autophagy

## Abstract

Cerebrospinal fluid (CSF) biomarkers play an important role in diagnosing Alzheimer’s disease (AD) which is characterized by amyloid-β (Aβ) amyloidosis. Here, we used two *App* knock-in mouse models, *App*^*NL-F/NL-F*^ and *App*^*NL-G-F/NL-G-F*^, exhibiting AD-like Aβ pathology to analyze how the brain pathologies translate to CSF proteomes by label-free mass spectrometry (MS). This identified several extracellular matrix (ECM) proteins as significantly altered in *App* knock-in mice. Next, we compared mouse CSF proteomes with previously reported human CSF MS results acquired from patients across the AD spectrum. Intriguingly, the ECM protein decorin was similarly and significantly increased in both *App*^*NL-F/NL-F*^ and *App*^*NL-G-F/NL-G-F*^ mice, strikingly already at three months of age in the *App*^*NL-F/NL-F*^ mice and preclinical AD subjects having abnormal CSF-Aβ42 but normal cognition. Notably, in this group of subjects, CSF-decorin levels positively correlated with CSF-Aβ42 levels indicating that the change in CSF-decorin is associated with early Aβ amyloidosis. Importantly, receiver operating characteristic analysis revealed that CSF-decorin can predict a specific AD subtype having innate immune activation and potential choroid plexus dysfunction in the brain. Consistently, in *App*^*NL-F/NL-F*^ mice, increased CSF-decorin correlated with both Aβ plaque load and with decorin levels in choroid plexus. In addition, a low concentration of human Aβ42 induces decorin secretion from mouse primary neurons. Interestingly, we finally identify decorin to activate neuronal autophagy through enhancing lysosomal function. Altogether, the increased CSF-decorin levels occurring at an early stage of Aβ amyloidosis in the brain may reflect pathological changes in choroid plexus, present in a subtype of AD subjects.

**Supplementary Information:**

The online version contains supplementary material available at 10.1186/s40478-022-01398-5.

## Introduction

Alzheimer’s disease (AD), as the most common cause of dementia, has two pathological hallmarks: extracellular amyloid-β peptide (Aβ) plaques and intracellular tau aggregation. Aβ could also accumulate in the vasculature and lead to changes in the blood–brain barrier (BBB) and in choroid plexus (ChP), which is part of blood-cerebrospinal fluid barrier (BCSFB), and potentially worsen disease progression [1–3]. The BCSFB is composed of endothelial cells, basement membrane and epithelial cells which form tight junctions that mediate selective transport of molecules and ions from blood during CSF production. Decreased CSF production has been reported in AD [4] due to dysfuncional BCSFB which may lead to imparied Aβ clearance from brain to the peripheral circulation, hence aggravating Aβ accumulation in the brain that further increases BBB dysfuntion [5]. During this vicious cycle, the basement membrane consisting of extracellular matrix (ECM) is affected. ECM is a highly dynamic network that consists of proteoglycans/glycosaminoglycans, collagen, elastin, fibronectin, laminins, and several other glycoproteins [6]. Already during the early stages of AD, significant changes of ECM effect brain microvascular function and may therefore drive AD progression [7]. Investigation of the CSF may give insights into AD brain pathology and a growing number of clinical research data support that the biomarkers of CSF play an important role in AD diagnostics [8, 9]. Three core CSF biomarkers, Aβ42, total-tau (t-tau) and phosphorylated-tau (p-tau), are used in the clinic to diagnose AD patients [10].

*App* knock-in mice exhibit AD-like phenotypes including robust Aβ pathology whereas *App* expression is at endogenous levels. *App*^*NL-F/NL-F*^ mouse model harbors two familial AD (FAD) mutations, the Swedish (NL) and the Beyreuther/Iberian (F), in the humanized mouse *App* gene which increases the human Aβ42 levels in the mouse brains [11]. The *App*^*NL-G-F/NL-G-F*^ mouse model has an additional Arctic mutation (G) leading to a much earlier and severer Aβ pathology which eventually cause more aggressive neuroinflammation, synaptic alteration and memory impairment [11].

In a translational approach to understand how pathological changes in the brain are mirrored in the CSF, we analyzed CSF of *App* knock-in mice by label-free mass spectrometry (MS) and compared their proteomes with protein changes in CSF of human subjects across the AD spectrum from a previously published large AD cohort [12]. We identified ECM protein decorin as similarly and significantly altered in CSF of three different AD mouse models exhibiting Aβ pathology and in preclinical AD subjects having abnormal-amyloid/normal-tau CSF levels (a + t −).

## Materials and methods

### Animals

The two *App* knock-in mouse models, *App*^*NL-F/NL-F*^ and *App*^*NL-G-F/NL-G-F*^, have been described previously [11]. Most of experiments related to these two *App* knock-in models were performed under ethical permit ID 12570-2021 approved by Stockholm animal ethical board and ID 407 approved by Linköping animal ethical board except the CSF collection from 13 months old *App*^*NL-F/NL-F*^ mice approved by RIKEN Center for Brain Science (W2021-2-020(2)). Two APP transgenic (Tg) mouse models were used, APP23 mice harboring only the Swedish mutation [13] (hemizygotes) and tg-ArcSwe mice containing both Swedish and Arctic mutations [14]. The CSF collection from APP23 mice were approved by Linköping animal ethical board (10925-2020). The CSF collection from tg-ArcSwe were approved by the Uppsala County animal ethics committee, the Swedish Board of Agriculture (5.8.18-20401/2020). Mice were kept on 12:12 light–dark cycle and with ad libitum access to food.

### Mouse CSF collection and brain dissection

The mice were anesthetized by isoflurane and placed on the heating pad with fixed heads (the head angle was around 135° from the body) on the stereotaxic instrument. The skin was incised sagittally after removal of the fur. Under the dissection microscope, subcutaneous tissues and muscles were separated to expose the dura mater of cisterna magna. To reduce blood contamination, the exposed dura mater was cleaned by PBS-soaked cotton swabs and punctured with a 27-gauge needle avoiding blood vessels. CSF was sampled with a glass capillary and collected in low affinity Eppendorf tubes and directly frozen. The collected CSF was discarded if there was any blood contamination under microscope inspection. Thereafter, the mice were perfused with PBS through cardiac perfusion, and hippocampus and cortex were dissected.

### Primary neuron culture

Twenty-four well plates were coated with Poly-D-lysine (Sigma-Aldrich, cat. P6407) for one hour at room temperature and washed with Milli-Q water followed by drying overnight. Embryos were separated from *App*^*wt/wt*^ mice E16-E18 and the heads were kept in HBSS (Thermo Fisher Scientific, Cat. 14175095) on ice for brain dissections. Brains were dissected under the dissection microscope to separate the cortex/hippocampus. Cortex/hippocampus were chopped and transferred to the falcon tube together with HBSS. HBSS was removed until only tissues were left at the bottom. Neurobasal medium (Thermo Fisher Scientific, Cat. 21103049) 97% + B-27 (Thermo Fisher Scientific, Cat. 17504044) 2% + Glutamax (Thermo Fisher Scientific, Cat. 35050038) 1% were added and tissues were separated by pipetting up and down 20–30 times. Cells were counted by a hematocytometer with trypan blue staining. 1.5 × 10^5^ cells for 24 well plates, 3 × 10^5^ cells for 12 well plates and 5 × 10^6^ cells for 10 cm petri dishes were seeded. After one week, 50% of the medium was replaced by a fresh medium.

For decorin treatment study, after 18 DIV, cells were treated with 200 nM recombinant mouse decorin (R&D Systems, Cat. 1060-DE-100) and/or 100 nM bafilomycin A1 (Sigma-Aldrich, Cat. B1793) for six hours and thereafter the cells were collected in PBS. Cell solutions were centrifuged at 1500 rpm for five min at 4 °C to pellet the cells, which were lysed in RIPA buffer (Thermo Fisher Scientific, Cat. 89901). The lysate was sonicated for one min and centrifuged at 15,000 rpm for 20 min at 4 °C. The supernatant was transferred to new tubes for western blot. Each condition was triplicated and the whole set of experiments repeated twice.

For human Aβ42 treatment study, after 12 DIV, cells were treated for 24 h with 50 nM recombinant Aβ42 (Met-Aβ42) which was prepared as described previously [15]. Then the cells were collected in RIPA buffer, sonicated for one min and centrifuged at 15,000 rpm for 20 min at 4 °C. The supernatant was transferred to new tubes for western blot. Each condition was repeated eight times and four out of eight were used for western blot analysis. Protein concentration was measured by BCA Protein Assay Kit (Thermo Fisher Scientific, Cat. 23225). SDS sample buffer containing 0.1 M dithiothreitol was loaded to the samples for western blot analysis.

### Immunohistochemistry

The paraffin-embedded mouse brains (12 months old) were sectioned into four µm thick sections for most of immunofluorescence staining except for vessel staining for which 10 µm thick sections was used. For decorin staining, slides were treated with 0.5 units of chondroitinase ABC (Sigma-Aldrich, Cat. C2905-10UN) in 0.1 M Tris-acetate (pH: 7.3) for four h in 37 °C before blocking [16]. For other antibodies, antigen retrieval was performed, whereas the combination of autoclaving and chondroitinase ABC treatment was performed for double staining. After blocking, slides were incubated with anti-Aβ antibody (82E1) (Immuno-Biological Laboratories, Cat.10323) (1: 2000), anti-decorin antibody (Thermo Fisher Scientific, Cat. PA513538) (single staining 1: 500, double staining 1: 50), anti-decorin antibody (R&D Systems, Cat. AF1060) (1: 200), anti-αSMA antibody (Sigma-Aldrich, Cat. F3777) (1: 500), anti-parvalbumin antibody (Sigma-Aldrich, Cat. MAB1572) (1: 4000) and anti-somatostatin antibody (Sigma-Aldrich, Cat. MAB354) (1: 100), for overnight at 4 °C respectively. The following day, slides were incubated with secondary antibodies: Biotinylated goat anti-Rabbit IgG (Vector Laboratories, Cat. BA-1000) (1: 200), Biotinylated goat anti-mouse IgG (Vector Laboratories, Cat. BA-9200) (1: 200), Biotinylated goat anti-rat IgG (Vector Laboratories, Cat. BA-9400) (1: 200), Alexa 546 goat anti-rabbit (Invitrogen, Cat. A11035) (1: 1000), Alexa 555 Donkey anti-goat (Invitrogen, Cat. A21432) (1: 1000). The biotinylated secondary antibodies were amplified with TSA Fluorescein System (PerkinElmer, Cat. NEL701001KT). Images were acquired by Nikon Eclipse E800 microscope with Nikon DS-Qi2 camera and quantified by ImageJ software.

### Western blot

The cell lysates were boiled at 95 °C for 3 min. 10–20 μg proteins were loaded onto 4–20% SDS-PAGE for separation and transferred to nitrocellulose membranes. The nitrocellulose membranes were blocked by 5% skim milk and were probed by primary antibodies, anti-p62 (Cell Signaling Technology, Cat. 5114) (1: 500), anti-LC3 (Novus Biologicals, Cat. NB100-2331) (1: 1000), anti-decorin (Invitrogen, Cat. PA5-13538) (1: 100), anti-LAMP1 (Abcam, Cat. ab24170) (1:500), anti-p-TFEB S142 (Merck, Cat. ABE1971) (1:200), anti-TFEB (Bethyl Laboratories, Cat. A303-673A) (1:200), anti-NBR1 (Proteintech, Cat. 16004-1-AP) (1:200), anti-β-actin (Sigma-Aldrich, Cat. A2228) (1: 10,000) overnight at 4 °C. Next day, the nitrocellulose membranes were incubated with fluorescently labeled secondary antibodies (LI-COR Biosciences), Donkey anti-rabbit (Cat. 926-32213) (1: 10,000) or Goat anti-mouse (Cat. 926-68070) (1: 10,000) for one hour at room temperature. Images were acquired by a fluorescence imaging system (LI-COR Biosciences, Odyssey CLx) and were analyzed by Image Studio Lite (LI-COR Biosciences) software.

### Phospho-explorer antibody array and analysis of biological processes

The phosphorylation profiling (Additional file 1: Table S1) of decorin-treated and non-treated mouse primary neurons was determined by Phospho Explorer Antibody Array (Full Moon BioSystems, Cat. PEX100) following the manufacturer’s protocol. The alterations of phospho-protein levels in decorin-treated/non-treated with ratios > 1.5 or < 0.6 were subjected to gene ontology enrichment analysis [17–19]. The biological processes with false discovery rate < 0.01 were considered as significantly enriched.

### Decorin enzyme-linked immunosorbent assay (ELISA) of mouse CSF

Mouse CSF decorin levels were measured by a decorin mouse ELISA kit (Abcam, Cat. ab155454) following the manufacturer’s protocol.

### Proximity extension assay

One μL of mouse CSF samples were applied to Mouse Exploratory Panel, Olink according to manufacturer’s protocol.

### Mass spectrometry analysis of mouse CSF

An aliquot of eight μL of each CSF sample was used for in-solution digestion. Briefly, the proteins were re-dissolved in 50 μL of digestion buffer (6 M urea, 100 mM TEAB). A volume of 10 μL of 45 mM aqueous dithiothreitol was added to all samples and the mixtures were incubated at 50 °C for 15 min to reduce cysteine disulfides. The samples were cooled to room temperature and 10 μL of 100 mM aqueous iodoacetamide was added before incubating the mixtures for an additional 15 min at room temperature in darkness to carbamidomethylate the cysteines. Finally, 10 μL of 0.1 μg/μL trypsin/Lys-C mixture dissolved in 50 mM ammonium bicarbonate was added to the samples. The tryptic digestion was performed at 37 °C overnight. Prior to MS analysis, the peptides were purified and desalted using SPE Pierce C18 Spin Columns (Thermo Fisher Scientific). The columns were activated by 2 × 200 μL of 50% acetonitrile and equilibrated with 2 × 200 μL of 0.5% trifluoroacetic acid. The tryptic peptides were adsorbed to the media using two repeated cycles of 40 μL sample loading and the column was washed using 3 × 200 μL of 0.5% trifluoroacetic acid. Finally, the peptides were eluted in 3 × 50 μL of 70% acetonitrile and dried. Dried peptides were resolved in 21 μL of 0.1% formic acid prior to nano-LC–MS/MS. The nano-LC–MS/MS experiments were performed using a Q Exactive Orbitrap mass spectrometer (Thermo Fisher Scientific, Bremen, Germany) equipped with a nano electrospray ion source. The peptides were separated by C18 reversed phase liquid chromatography using an EASY-nLC 1000 system (Thermo Fisher Scientific). A set-up of pre-column and analytical column was used. The precolumn was a 2 cm PepMap Acclaim (C18 100 µm, 5 µm particles) (Thermo Fisher Scientific) while the analytical column was a 10 cm EASY-Spray column (C18 75 µm, 3 µm particles, Thermo Fisher Scientific). Peptides were eluted with a 150 min linear gradient from 4 to 100% acetonitrile at 250 nL min-1. The mass spectrometer was operated in positive ion mode, acquiring a survey mass spectrum with resolving power 70,000 (full width half maximum), m/z 400–1750 using an automatic gain control target of 3 × 10^6^. The 10 most intense ions were selected for higher-energy collisional dissociation fragmentation (25% normalized collision energy) and MS/MS spectra were generated with an automatic gain control target of 5 × 10^5^ at a resolution of 17,500. The mass spectrometer was operated in data-dependent mode. Acquired raw files were processed by MaxQuant (version 1.5.1.2) (the software is available at http://www.maxquant.org). Tandem mass spectra were searched with Andromeda against the UniProt Mus musculus database (release January 2017). The search settings were set as: maximum 10 ppm and 0.02 Da error tolerance for the survey scan and MS/MS analysis respectively; enzyme specificity was trypsin/Lys-C; maximum two missed cleavage sites were allowed; cysteine carbamidomethylation was set as static modification, and Oxidation (M) was set as dynamic modification.

The search criteria for protein identification were set to at least two matching peptides. No proteins were identified and quantified using only one peptide. A maximum false discovery rate of 1% for peptides and proteins was selected. Both razor and unique peptides were used for quantification. For decorin, at least five peptides per sample were used for the quantification (Additional file 2: Table S2). A decoy sequence database was built by reversing the target sequence database. A list of known contamination was also included in the identification. The protein intensity values were used for further data analysis.

### Mass spectrometry analysis of human CSF

CSF proteomic results reported in a previous study were included in the present research [12]. Briefly, we selected individuals with AD pathology defined as abnormal CSF-Aβ42, and we subdivided this group into abnormal (a + t + , n = 151) and normal CSF-t-tau groups (a + t −, n = 77). Based on their cognitive performance, AD individuals were classified in three clinical stages as preclinical AD (normal cognition, i.e., NC), prodromal AD (mild cognitive impairment, i.e., MCI) and mild to moderate AD-type dementia according to study specific criteria [20]. MS was performed using the tandem mass tag (TMT) technique with 10 + 1 multiplexing as previously described, and high-pH reverse phase HPLC for peptide prefractionation [12, 21]. The EMIF-AD MS proteomics data have been deposited to the ProteomeXchange Consortium via the PRIDE partner repository with the dataset identifier 10.6019/PXD019910 [22]. Fold-changes were computed for a + t − and a + t + groups according to a control group (n = 82) with intact cognition and normal CSF amyloid and tau markers.

### Comparison of mouse and human CSF proteomes and analysis of biological processes

The same protein identifiers were used to compare mouse and human CSF proteomes. We performed two types of comparisons. In the first exploratory comparison, proteins with fold change > 1.0 was considered to be upregulated whereas < 1.0 was considered downregulated in the mouse and human CSF proteomes. In the second comparison only signifcantly changed proteins in the mouse and human CSF samples (*p* < 0.05) were compared.

### Statistical analysis

For the analysis of mouse CSF proteomics data, protein intensities were first transformed to log2 scale and a two-tailed Student’s t-test was performed on proteins which were identifed in all samples in order to identify proteins that were significantly altered in *App* knock-in mouse models, using Qlucore software. A *p* < 0.05 was considered as statistically significant. Venn diagrams were generated by the Interactive Venn tool [23] or Venny 2.1 [24]. To investigate the variation among individual mouse CSF proteomes, a principal component analysis (PCA) was performed in Qlucore. Volcano plots were generated by GraphPad Prism 8 and significantly altered proteins in *App* knock-in models, *p* < 0.05, were indicated. In order to visualize the significantly altered proteins in *App* knock-in mice, heat maps were generated by Morpheus software from the Broad Institute [Morpheus, https://software.broadinstitute.org/morpheus]. For the analysis of biochemical data, one-way ANOVA followed by Dunnett’s multiple comparisons test was performed for the three group comparisons or more while two tailed student’s t-test was performed for the two-group comparisons in GraphPad Prism 8 when the datasets were normally distrubuted. If the dataset was not normally distributed, non-parametric Kruskal–Wallis tests with Dunn’s post hoc analysis was used. Decorin distribution in different cell types in mouse hippocampus were analyzed by two-way ANOVA followed by Tukey’s post hoc test.

CSF-decorin levels across the AD spectrum were analyzed by one-way ANOVA followed by Tukey’s multiple comparisons test. Associations between human CSF-decorin levels (outcome) and CSF Aβ42, t-tau and p-tau levels (predictors) were determined using linear regression across the total group, and differences between subgroups (i.e., a − t −, a + t −) in NC group were tested with the ‘emtrends’ function from the emmeans R package (v1.5.2). ROC curve analyses were performed for CSF-decorin levels and AUC with 95% CI were calculated with R package pROC (v1.18.0).

The correlation analysis between parameters of Aβ pathology and decorin expression in the mice were made by Spearman’s rank correlation coefficient since some of the parameters were not normally distributed. The data were grouped from *App*^*wt/wt*^ and *App*^*NL-F/NL-F*^ mice and *App*^*wt/wt*^ and *App*^*NL-G-F/NL-G-F*^ mice, respectively.

## Results

**Fig. 1:**
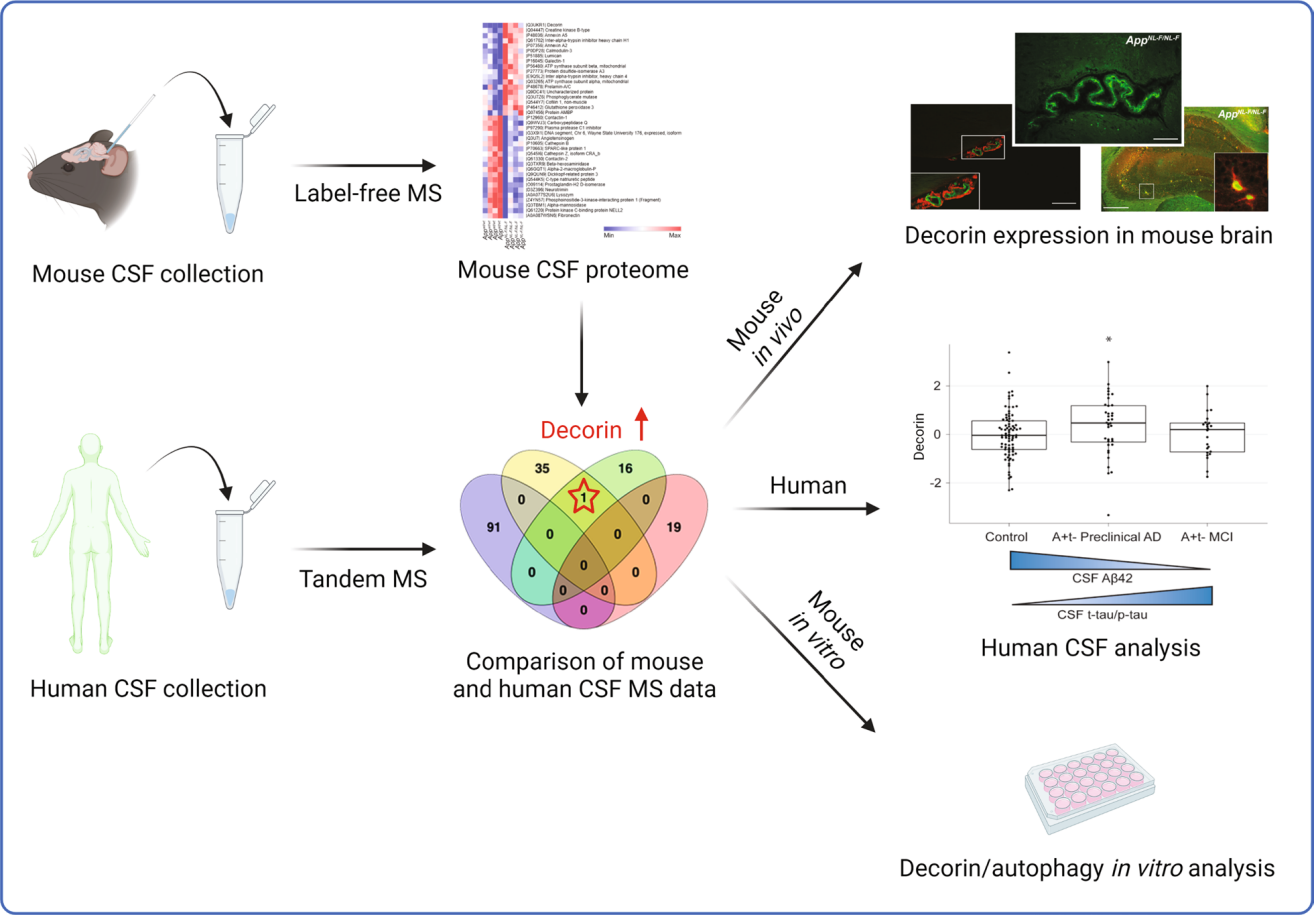
Schematic representation of the study using mouse and human CSF. *CSF* cerebrospinal fluid, *MS* mass spectrometry

To investigate how AD pathologies driven by Aβ translate to CSF, we used label-free MS to analyze the CSF of *App* knock-in mice which exhibit robust Aβ amyloidosis. To stratify the data, the mouse CSF proteomes were next compared with the proteomes of human CSF from a previously published large AD cohort [12]. This resulted in identification of protein hits related to ECM and autophagy which were followed up and investigated by biochemical means. The workflow of the whole project is presented in Fig. 1.

### ECM proteins are altered in CSF of *App* knock-in mice

**Fig. 2:**
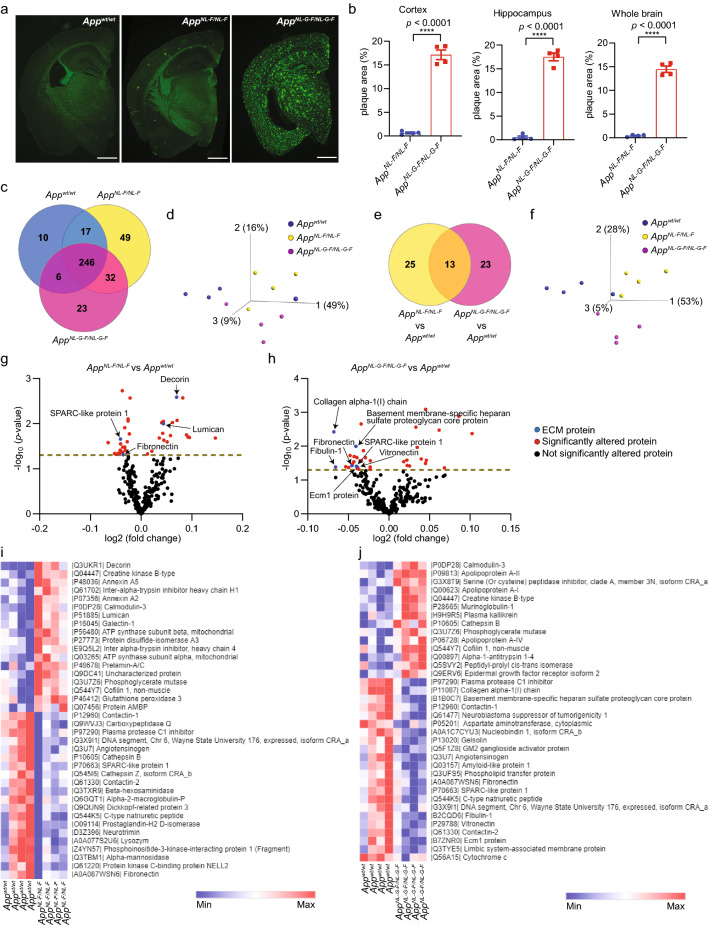
Mass spectrometry analysis of CSF from *App* knock-in mice identifies changes in ECM proteins. **a**, **b** Immunostaining and quantification of Aβ plaques in 12 months old *App* knock-in mouse brains. Scale bars, 1000 µm. (n = 4). **c** Number of proteins identified in CSF of the same mice and **d** PCA of proteins identified in all samples. (n = 4). **e**, **f** Number and PCA of significantly (*p* < 0.05) altered proteins. **g** Volcano plots displaying protein changes in *App*^*NL-F/NL-F*^ and (h) *App*^*NL-G-F/NL-G-F*^ mice. Dash line: *p* = 0.05. **i** Heatmaps showing significantly (*p* < 0.05) changed proteins in *App*^*NL-F/NL-F*^ mice and **j** *App*^*NL-G-F/NL-G-F*^ mice. Proteins were sorted into upregulated and downregulated in the *App* knock-in mice and by significance from highest to lowest (top to bottom). Data in (**b**) were analyzed by student’s t-test. Data are represented as mean ± SEM; ****p < 0.0001

In a translational approach to identify potential AD-related CSF biomarkers induced by Aβ and its downstream effects, we investigated the protein changes in CSF of 12 months old *App*^*NL-F/NL-F*^ and *App*^*NL-G-F/NL-G-F*^ mice which exhibit different degree of Aβ pathology (Fig. 2a, b). Taking the limited volume of mouse CSF into account, a label-free MS approach was used which identified 427–703 proteins of which 246 were detected in all samples and their levels quantified (Fig. 2c; protein list in Additional file 2: Table S2). Principal component analysis (PCA) of these 246 proteins indicated a separation between the three groups (Fig. 2d) whereas PCA of the significantly altered proteins (*p* < 0.05) resulted in a tighter clustering (Fig. 2e, f). Interestingly, ECM proteins decorin and lumican were upregulated in *App*^*NL-F/NL-F*^ mice while SPARC-like protein 1 and fibronectin were downregulated (Fig. 2g, i; Additional file 3: Table S3). However, in *App*^*NL-G-F/NL-G-F*^ mice, most of ECM proteins were downregulated including collagen alpha-1(I) chain, basement membrane-specific heparan sulfate proteoglycan core protein, fibronectin, SPARC-like protein 1, fibulin-1, vitronectin and ecm1 protein (Fig. 2h, j; Additional file 3: Table S3). Comparing the CSF proteomes of the two *App* knock-in mouse models directly with each other revealed significant (*p* < 0.05) changes in additional ECM related proteins (Additional file 4: Fig. S1a, b; Additional file 3: Table S3). Several studies have previously found that the dysfunction of BBB and BCSFB, including changes in ECM proteins, increase upon aging and in AD [25–27] and we therefore also analyzed the CSF of 18 months old *App* knock-in mice (n = 5) using a mouse proximity extension assay (PEA) panel. Indeed, two ECM proteins, matrilin-2 and CCN family member 4, were significantly (*p* < 0.05) altered (Additional file 3: Table S3, Additional file 5: Table S4). Altogether, several ECM proteins were significantly altered in CSF of the *App* knock-in mice, and they are differently altered depending on the severity of the brain Aβ pathology.

### Decorin is similarly increased in CSF of both ***App***^***NL-F/NL-F***^ mice and preclinical AD subjects with abnormal CSF-Aβ42

In an attempt to further understand how changes in the CSF proteomes of *App* knock-in mouse models reflect those observed in human patients, we next compared them to MS-characterized CSF proteomes of a large human European Medical Information Framework for Alzheimer’s Disease Multimodal Biomarker Discovery (EMIF-AD MBD) cohort (n = 310) [12]. In this cohort (clinical data for the patients is summarized in Additional file 6: Table S5), individuals with abnormal CSF-Aβ42 were classified into three clinical stages; preclinical AD (normal cognition, i.e., NC), prodromal AD (mild cognitive impairment, i.e., MCI) and mild to moderate AD-type dementia, based on their cognitive performance [20]. The groups were further classified according to CSF-t-tau levels, being normal (a + t −) or abnormal (a + t +). The CSF proteome alterations in the NC, MCI and AD groups were based on the comparison to the healthy control subjects having normal CSF-Aβ42 and CSF-tau, and normal cognition. Interestingly, the number of proteins with a relative expression level below one in both the *App* knock-in mice and a + t − human subjects were higher as compared to a + t + subjects, indicating that the alterations in the CSF proteomes of *App* knock-in mice are, at least to some extent, more similar to the CSF alterations observed in a + t − human subjects (Table 1, Additional file 7: Table S6, Additional file 8: Table S7). This may reflect that *App* knock-in mice have a strong Aβ pathology whereas the tau pathology is less pronounced [28]. Further mouse and human CSF proteome comparisons were therefore performed with a + t − human subjects.

**Table 1:** Number of proteins with expression level above one or below one in *App* knock-in mice and in human cohorts

	Abnormal-amyloid/normal-tau						Abnormal-amyloid/abnormal-tau					
	and *App*^*NL-F/NL-F*^			and *App*^*NL-G-F/NL-G-F*^			and *App*^*NL-F/NL-F*^			and *App*^*NL-G-F/NL-G-F*^		
	NC	MCI	AD	NC	MCI	AD	NC	MCI	AD	NC	MCI	AD
Number of proteins expression level above one	33	46	35	38	50	37	39	47	53	38	47	53
Number of proteins expression level below one	76	84	81	84	90	86	24	26	27	26	28	29

A detailed list of proteins and comparisons are provided in table S6 and S7

**Fig. 3:**
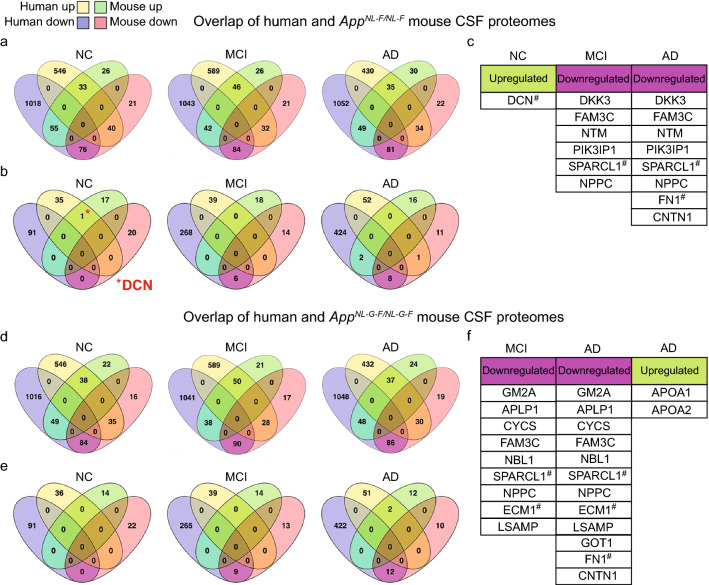
Decorin is significantly increased in CSF of *App*^*NL-F/NL-F*^ mice and a + t− preclinical AD subjects. **a** Venn diagrams showing the number of proteins with expression levels above one and below one and **b** significantly changed proteins (*p* < 0.05) in the CSF of *App*^*NL-F/NL-F*^ mice and NC/MCI/AD human cohorts from abnormal-amyloid/normal-tau subjects. Among those significantly changed proteins, ECM protein decorin is the only significantly upregulated protein in NC subjects and *App*^*NL-F/NL-F*^ mice. **c** The gene names of significantly upregulated and downregulated proteins in both *App*^*NL-F/NL-F*^ mouse and human CSF. **d** Venn diagrams showing the number of proteins with expression levels above one and below one and **e** significantly changed proteins (*p* < 0.05) in the CSF of *App*^*NL-G-F/NL-G-F*^ mice and NC/MCI/AD human cohorts from abnormal-amyloid/normal-tau subjects. **f** The gene names of significantly upregulated and downregulated proteins in both *App*^*NL-G-F/NL-G-F*^ mouse and human CSF. ^#^ denotes ECM-associated proteins. *NC* normal cognition, *MCI* mild cognitive impairment, *AD* mild to moderate AD-type dementia, *CSF* cerebrospinal fluid, *DCN* decorin

A qualitative direct comparison of CSF MS data from a + t − NC subjects and *App*^*NL-F/NL-F*^ mice showed that 33 proteins exhibited relative expression levels above one (Fig. 3a). Notably, among those, only decorin (*DCN)* was significantly upregulated in both *App*^*NL-F/NL-F*^ mice and a + t − NC subjects (*p* < 0.05) (Fig. 3b). The comparison of *App*^*NL-F/NL-F*^ mice and a + t − MCI and AD subjects revealed two significantly downregulated ECM proteins SPARC-like protein 1 (*SPARCL1*) and fibronectin (*FN1*) (Fig. 3c). Comparing *App*^*NL-G-F/NL-G-F*^ mouse CSF proteome with the human CSF proteome revealed significant downregulation of ECM proteins *SPARCL1, FN1* and ecm1 protein (*ECM1*) (Fig. 3d–f). Taken together, several ECM proteins were significantly and commonly altered in mouse and human CSF proteomes and among those, decorin is increased already at an early stage of the Aβ pathology in both *App*^*NL-F/NL-F*^ mice and a + t − NC subjects.

### CSF-decorin positively correlates with CSF-Aβ42 in a + t − preclinical AD subjects and predicts a subtype of AD

**Fig. 4:**
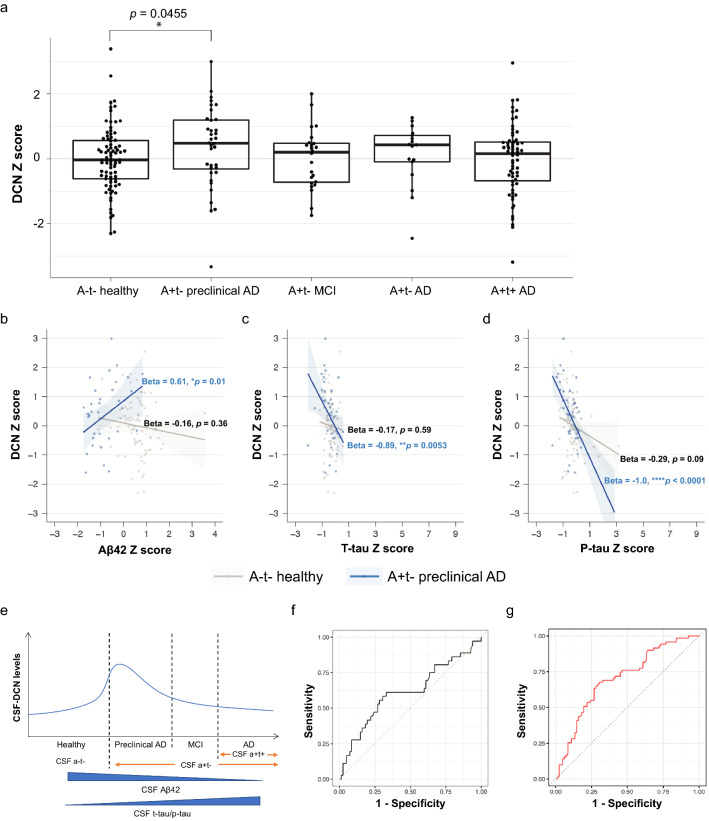
CSF-decorin positively correlates with CSF-Aβ42 in a + t− preclinical AD and predicts a subtype of AD. **a** CSF-DCN levels in a-t- healthy (n = 82) and a + t− subjects with preclinical AD (n = 36), MCI (n = 24), AD (n = 17), and a + t + AD (n = 62). (*p < 0.05 as compared to a-t- healthy). **b** Correlations of CSF-DCN and CSF-Aβ42, **c** CSF-DCN and CSF-t-tau, **d** CSF-DCN and CSF-p-tau in a-t− healthy and a + t− preclinical AD subjects. The regression coefficients (Beta) and *p*-values are displayed. (*p < 0.05, **p < 0.01, ****p < 0.0001). **e** Hypothetical curve showing CSF-DCN alteration from a-t− healthy subjects to a + t− preclinical AD, MCI, AD and a + t + AD subjects and its correlation with CSF-Aβ42 and CSF-tau levels. **f** Receiver operating characteristic (ROC) analysis was performed in a + t- preclinical AD (n = 36) versus healthy control (n = 82) and revealed an area under the ROC Curve (AUC) (95% confidence interval (CI)) = 0.61 (0.50, 0.73) (*p* = 0.058) for CSF-DCN levels. **g** ROC curve was performed in a subtype classified by as innate immune activation (n = 71) versus healthy control (n = 82) and revealed an AUC (95% CI) = 0.70 (0.62, 0.79) (*p* = 1.73 × 10^–6^) for CSF-DCN levels. Data in (**a**) were analyzed by one-way ANOVA followed by Tukey’s multiple comparisons test. Data are represented as mean ± SEM. DCN: decorin

Having found that decorin is specifically increased in CSF of a + t − NC subjects but not in a + t− MCI and AD subjects suggested an association of decorin to early AD pathologies and hence be of interest as a future potential biomarker (Fig. 4a). Therefore, we next investigated how decorin levels were related to the AD CSF biomarkers Aβ42, t-tau and p-tau in human CSF. Within the a − t − healthy subjects CSF-decorin levels followed a non-linear trajectory with CSF-Aβ42 (beta(se) = -0.16(0.18), *p* = 0.36). In contrast, within a + t − NC subjects CSF-decorin levels significantly correlated with CSF-Aβ42 levels (beta(se) = 0.61(0.25), *p* = 0.01), a correlation that was significantly (*p* = 0.016) different from that of a-t- healthy subjects (Fig. 4b). In other words, in healthy subjects with normal levels of CSF-Aβ42, CSF-decorin levels tend to increase with decreasing CSF-Aβ42 levels until CSF-Aβ42 reaches pathological levels. When CSF-Aβ42 levels reach abnormal levels, the correlation switches and becomes positive. That is, within the group of a + t − preclinical AD subjects that have abnormal CSF-Aβ42 levels, the CSF-decorin levels start to decrease (Fig. 4e). In addition, plotting CSF-decorin against CSF-t-tau and CSF-p-tau within the a-t− healthy group revealed non-linear associations between CSF-decorin and CSF-t-tau or CSF-p-tau. In contrast, the correlations became significantly negative in a + t − NC subjects for CSF-decorin and CSF-t-tau (beta(se) = − 0.89(0.32), *p* = 0.0053) (Fig. 4c) and CSF-decorin and CSF-p-tau (beta(se) = − 1.00(0.21), *p* < 0.0001) (Fig. 4d). Furthermore, across the whole disease spectrum including NC, MCI and AD subjects (n = 310), lower CSF-decorin levels correlated to higher CSF-t-tau (beta(se) = − 0.11(0.04), *p* = 0.0014) (Additional file 9: Fig. S2b) and CSF-p-tau levels (beta(se) = − 0.16(0.04), *p* < 0.0001) (Additional file 9: Fig. S2c) but no association with CSF-Aβ42 levels was observed (beta(se) = 0.09(0.06); *p* = 0.11) (Additional file 9: Fig. S2a) which is expected considering that decorin increases early in the a + t − preclinical AD subjects and then subsequently decreases upon disease progression (Fig. 4e). We next explored whether increase of CSF-decorin levels might have use as a biomarker for early Aβ amyloidosis by performing receiver operating characteristic (ROC) analysis in a + t − preclinical AD subjects *vs* healthy control. The area under the curve (AUC) was 0.61 (95% confidence interval (CI) = 0.50, 0.73) and not significant within this rather limited cohort (*p* = 0.058) (Fig. 4f). However, we previously observed distinct AD subtypes showing non-linear alterations in CSF proteomics, which may weaken decorin relationships with AD if not taken into account [12]. One subtype (including all the subjects in preclinical AD, MCI and AD) was characterized by innate immune activation, specific increases in proteins associated with ECM and increased transthyretin, which may indicate ChP dysfunction. Interestingly, CSF-decorin alterations could very well classify these subjects from healthy control with an AUC (95% CI) = 0.70 (0.62, 0.79) (*p* = 1.73 × 10^–6^) (Fig. 4g). Taken these data together indicate that the increase of CSF-decorin levels may reflect the innate immune activation and potentially ChP dysfunction in the brain.

### Increased CSF-decorin in three different mouse models of Aβ pathology

**Fig. 5:**
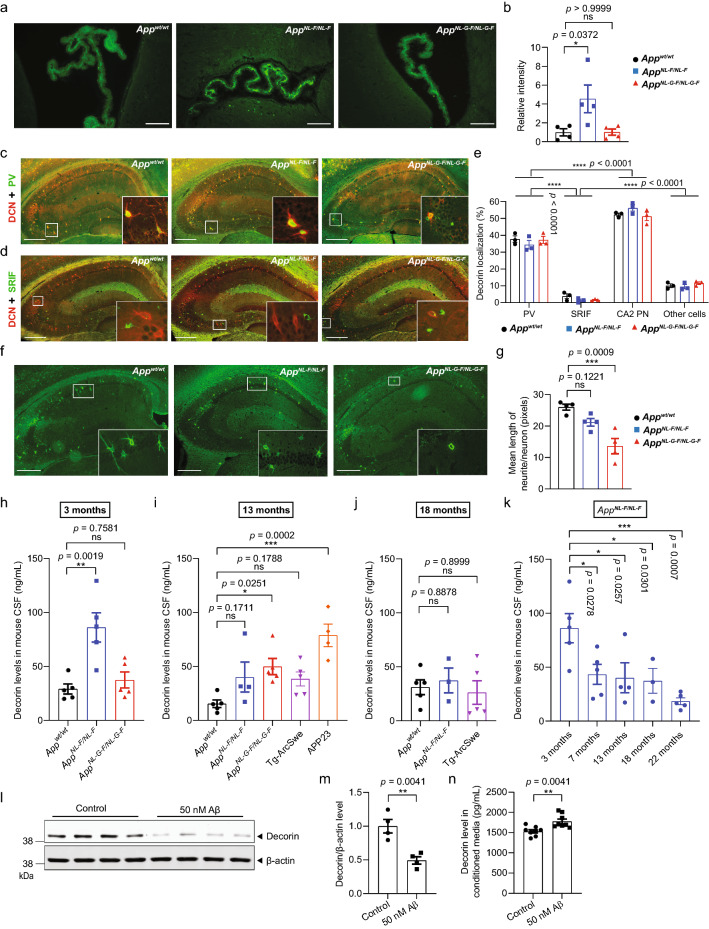
Increased CSF-decorin in three different mouse models of Aβ pathology. **a**, **b** Immunostaining and quantification of decorin in the ChP. Scale bars, 100 µm. (n = 4). **c** Double immunostaining of DCN and PV or **d** SRIF in mouse hippocampus. **e** Quantification of decorin-positive cell-type distribution. Scale bars, 500 µm. (n = 3). **f**, **g** Immunostaining and quantification of decorin in the hippocampus. Scale bars, 500 µm. (n = 4). **h** Mouse CSF-decorin levels in three months old (n = 5), **i** 13 months old (n = 4–5), **j** 18 months old (n = 3–5) mice were measured by ELISA and quantified. **k** CSF-decorin levels in *App*^*NL-F/NL-F*^ mice of different ages were measured and quantified. (n = 3–5). **l** Decorin levels in Aβ42 treated mouse primary neurons and (**m**) were quantified (n = 4). **n** Quantification of decorin levels in conditioned media. (n = 8). Data in (**b**, **g**–**k**) were analyzed by one-way ANOVA followed by Dunnett’s multiple comparisons test. Data in (**m**, **n**) were analyzed by student’s t-test. Data in (**e**) were analyzed by two-way ANOVA followed by Tukey’s post hoc test. Data are represented as mean ± SEM; *p < 0.05, **p < 0.01, ***p < 0.001, ****p < 0.0001. *DCN* decorin, *PV* parvalbumin, *SRIF* somatotropin release-inhibiting factor, *PN* pyramidal neurons, *ns* not significant

Having found that decorin was increased in CSF proteome of *App*^*NL-F/NL-F*^ mice, we were interested in the potential decorin alterations in the same mouse brains as used for CSF analysis. Interestingly, decorin expression was significantly higher in ChP in *App*^*NL-F/NL-F*^ mice (Fig. 5a, b). Decorin were also localized in neurons in both cortex and hippocampus wherein decorin was highly expressed in CA2 pyramidal neurons and parvalbumin (PV)-positive interneurons, but little expressed in somatotropin release-inhibiting factor (SRIF)-positive interneurons (Fig. 5c–e). Interestingly, the neurites of PV-interneurons in *App* knock-in mice, especially in the *App*^*NL-G-F/NL-G-F*^ mice, contained significantly less decorin (Fig. 5f, g). In addition, decorin was highly expressed in the tunica externa of arteries (Additional file 10: Fig. S3a) and veins (Additional file 10: Fig. S3b) on the surface of the brain but not present in the vessels of brain parenchyma (Additional file 10: Fig. S3c). However, no significant differences in decorin levels were observed in the vessels (Additional file 10: Fig. S3d, e). Summarizing the decorin alterations in CSF and in the brains of *App* knock-in mice by Spearman’s correlation analysis, revealed that the increase of CSF-decorin positively correlated both with decorin levels in ChP and Aβ plaque load in *App*^*NL-F/NL-F*^ mice (Additional file 11: Fig. S4a) and that the reduced decorin expression in the interneurons was associated with the severe Aβ amyloidosis in *App*^*NL-G-F/NL-G-F*^ mice (Additional file 11: Fig. S4b).

To deepen the understanding of the relationship of decorin and the progression of Aβ amyloidosis, we measured the CSF-decorin levels in *App* knock-in mice of different ages as well as in two additional APP tg mouse models exhibiting Aβ pathology; APP23 and tg-ArcSwe, which have the same genetic background (C57BL/6J) as the *App* knock-in mice, by enzyme-linked immunosorbent assay (ELISA). APP23 mice contain only the Swedish mutation and form Aβ plaques from around 12 months of age, while tg-ArcSwe mice having both the Swedish and the Arctic mutation start to form Aβ plaques at around six months of age [29]. Notably, a significant increase of CSF-decorin levels were detected already in three months old *App*^*NL-F/NL-F*^ mice (Fig. 5h). At 13 months of age, the CSF-decorin levels were significantly increased in both *App*^*NL-G-F/NL-G-F*^ and APP23 mice while there was a non-significant trend towards increased levels in *App*^*NL-F/NL-F*^ and tg-ArcSwe mice (Fig. 5i). The CSF-decorin levels in 18 months old *App*^*NL-F/NL-F*^ and tg-ArcSwe mice were not altered (Fig. 5j). Since we found increased CSF-decorin levels in the early preclinical stage of AD, we were interested in the CSF-decorin changes in the *App*^*NL-F/NL-F*^ mice exhibiting a mild Aβ pathology and how it is changed upon development of the Aβ amyloidosis. Therefore, we deepened the analysis of the *App*^*NL-F/NL-F*^ mice and measured the CSF-decorin levels in 3, 7, 13, 18, 22 months of age. Interestingly, we found a clear decrease in the aged mice after the initial increase similar to the human CSF-decorin alterations (Fig. 5k). To investigate the causal relationship between decorin and Aβ42, we treated *App*^*wt/wt*^ mouse primary neurons with 50 nM of recombinant human Aβ42. This led to significantly decreased levels of decorin in the neurons (Fig. 5l, m), whereas the decorin levels in the conditioned media were significantly increased (Fig. 5n). This indicated that a low concentration of Aβ42 increased the neuronal secretion of decorin. Taken together, CSF-decorin levels are increased in both *App* knock-in and APP23 mice exhibiting Aβ amyloidosis, and the levels go down upon increased Aβ42 pathology in the brain, similar to the CSF-decorin alterations in a + t − human subjects.

### Decorin activates autophagy-lysosomal pathway by modulating lysosomal degradation

**Fig. 6:**
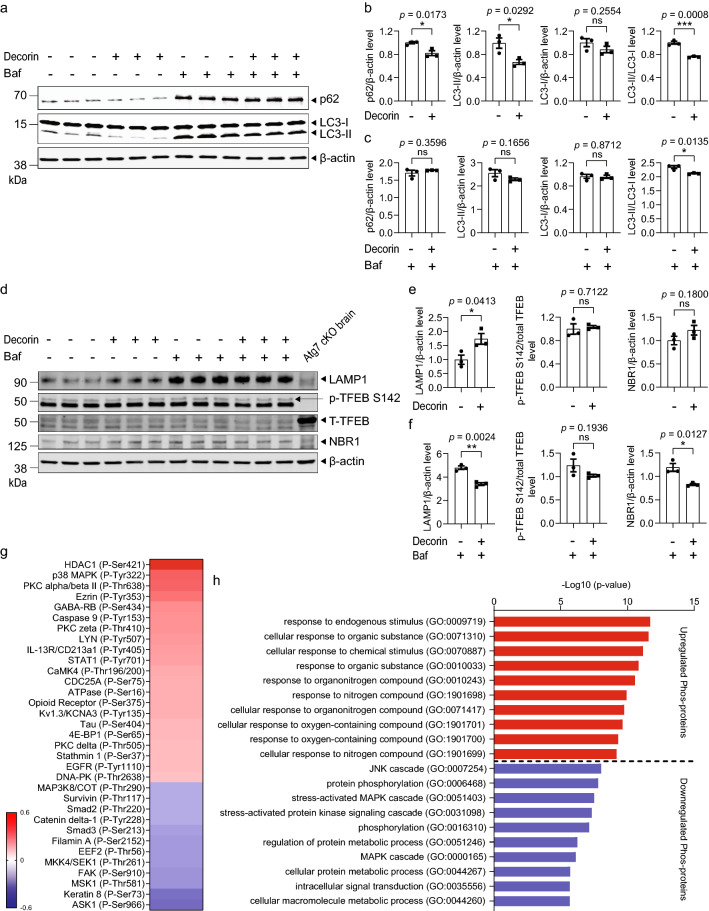
Decorin activates autophagy-associated lysosomal degradation. **a**–**c** Quantitative western blot of p62 and LC3 levels in mouse primary neurons treated with decorin and/or bafilomycin A1. **d**–**f** Quantitative western blot of LAMP1, p-TFEB S142, T-TFEB and NBR1 levels in mouse primary neurons treated with decorin and/or bafilomycin A1. Brain homogenate from *Atg7* conditional knockout mouse was loaded in the right most lane. **g** Phosphorylation profiling of proteins with ratios greater than 1.5 and lower than 0.6 comparing decorin-treated to non-treated. Scale bars, log10 of the ratio. **h** The 34 phospho-proteins identified in (**g**) were separately uploaded to GO and top 10 significantly enriched GO terms of biological processes are shown. (False discovery rate < 0.01). Data in (**b**, **c**, **e**, **f**) were analyzed by student’s t-test. Data are represented as mean ± SEM; *p < 0.05, ***p < 0.001; ns: not significant. *Baf* bafilomycin A1, *p-TFEB S142* phosphorylated TFEB Ser142, *T-TFEB* total TFEB

Previous study has shown that decorin activates autophagy in endothelial cells [30] which prompted us to investigate the relationship between decorin and autophagy in a neuronal setting. Interestingly, treating *App*^*wt/wt*^ mouse primary neurons with decorin decreased the level of p62, microtubule associated protein 1 light chain 3 (LC3)-II and LC3-II/LC3-I ratio (Fig. 6a, b). However, co-treatment with bafilomycin A1 (an inhibitor of lysosomal proteolysis) and decorin led to no change of LC3-II and p62 levels but to a decrease of LC3-II/LC3-I levels as compared to bafilomycin A1 treatment alone (Fig. 6a, c). This indicates that decorin may stimulate autophagy flux by enhancing downstream lysosomal degradation rather than by increasing initiation of autophagy. The levels of lysosomal-associated membrane protein 1 (LAMP1), a marker for the amount and integrity of lysosomes, were significantly increased in decorin-treated neurons whereas the levels of a master regulator of the lysosome biogenesis, transcription factor EB (TFEB), were not changed (Fig. 6d, e). Interestingly, the LAMP1 levels were decreased by co-treatment of decorin and bafilomycin A1 as compared to the single bafilomycin A1 treatment which indicates that decorin may ameliorate the lysosomal inhibition (Fig. 6d, f), which is additionally supported by a decrease in the levels of autophagy receptor NBR1 (Fig. 6d, f). These data demonstrate that decorin influences the lysosomal degradation and potentially rescues partially the lysosomal inhibition by bafilomycin. The analysis of the decorin signaling pathways revealed no significant changes of phospho-proteins associated with autophagy upstream pathways (Additional file 12: Fig. S5). However, several proteins (apoptosis signal-regulating kinase 1 (ASK1), SAPK/Erk kinase (MKK4/SEK1), focal adhesion kinase (FAK), mothers against decapentaplegic homolog 3 (Smad3)) related to c-Jun N-terminal kinases (JNK)/mitogen-activated protein kinase (MAPK) signaling pathways exhibited significantly altered phosphorylation status (Fig. 6g, h) which play a role in autophagy regulation [31].

## Discussion

In this study we aimed at determining how AD brain pathologies are reflected in CSF and their translational potential toward clinical use, using data from both AD mouse models, and human subjects. Here, we used two *App* knock-in mouse models exhibiting different degree of Aβ pathology, and analyzed their CSF by label-free MS. This revealed alterations in several ECM related proteins, including decorin and lumican that were specifically increased in the CSF of *App*^*NL-F/NL-F*^ mice. Interestingly, decorin and lumican are highly expressed in the recently identified vascular cell type fibroblast-like cells which reside within the perivascular Virchow–Robin space in the mouse brain [32]. The perivascular pathway facilitates CSF flow and enhances the clearance of Aβ [33]. Most importantly, decorin was similarly and significantly increased in CSF from both *App*^*NL-F/NL-F*^ mice and a + t − NC human subjects. In addition, an increase of CSF-decorin is also found in APP Tg mice exhibiting Aβ amyloidosis, further generalizing the correlation of Aβ amyloidosis and CSF-decorin. Notably, among a + t − NC subjects, a significant positive correlation of CSF-decorin and the well-established biomarker CSF-Aβ42 was found, and the correlation was significantly switched from the non-linear negative correlation in the a-t− healthy subjects. In other words, among a − t − healthy subjects that are on the trajectory towards preclinical AD, the CSF-decorin levels transiently increases as CSF-Aβ42 decreases. After reaching a peak in CSF-decorin levels in a + t − preclinical AD subjects, CSF-decorin levels start to decrease along with a continuous decrease in CSF-Aβ42 levels, and in a + t − MCI subjects the CSF-decorin levels are back to that of a-t-healthy subjects. Hence, a change in CSF-decorin level is associated with an early Aβ pathology or even increased levels of soluble Aβ species. This transient increase in CSF-decorin followed by a subsequent decrease may explain that CSF-decorin and CSF-Aβ42 do not correlate over the whole disease spectrum. In addition, CSF-Aβ42 levels are low already at an early stage of AD and continue to be stable during the AD dementia stage [34]. The ROC analysis of the a + t − preclinical AD subjects (n = 36) showed that CSF-decorin levels could not classify this group with high accuracy (AUC = 0.061, *p* = 0.058). However, decorin could indeed classify with high sensitivity and specificity a subtype of CSF-amyloid positive subjects including NC, MCI, AD and whose CSF proteome was characterized by innate immune activation, increased ECM proteins and increased transthyretin indicating a potential ChP dysfunction (AUC = 0.70, *p* = 1.73 × 10^–6^). Further analysis of ChP pathology in AD brains of this AD subtype and its correlation with CSF-decorin is warranted. Moreover, the CSF-decorin levels significantly negatively correlated with both CSF-t-tau and CSF-p-tau in a + t − NC subjects as well as over the whole AD spectrum. This additionally supports the link of decorin and the tau pathology in AD brain which needs to be addressed in mouse models of tau pathology.

A growing number of studies have shown that BBB breakdown occurs in early AD and is closely related to cognitive dysfunction [35–37]. Hence, finding a potential CSF biomarker, such as decorin, for the early changes of BBB would be suitable for predicting the early changes of cognition. In our study we additionally found other ECM proteins including SPARC-like protein 1, fibronectin and ecm1 protein to be significantly downregulated in a similar manner in *App* knock-in mouse models and a + t − MCI and AD human subjects showing that such changes are translational between mice and human. Considering that ECM is an important constituent of BBB/BCSFB, which are most likely affected in AD [2, 4], our proteomic findings suggest that the *App* knock-in mice could also have changes in the BBB/BCSFB.

Decorin has been identified in Aβ depositions in AD brains [16] whereas a disturbed decorin expression is observed in fibroblasts from sporadic AD patients [38]. Our finding that Aβ42 induces secretion of decorin from neurons may explain the decreased level of decorin in PV-positive interneurons in hippocampus. On the other hand, it cannot be ruled out that decorin alterations in other cell types *e.g.*, glia cells, induced by Aβ amyloidosis may eventually lead to CSF-decorin changes. This needs to be further investigated. Furthermore, we showed that decorin expression in ChP was increased in *App*^*NL-F/NL-F*^ mice which correlated both with Aβ pathology and elevated CSF-decorin level. Taking these data together indicates that early Aβ amyloidosis, potentially involving soluble Aβ species, in the brain may increase the decorin expression in ChP which may also lead to elevated decorin levels in the CSF since CSF is produced in ChP. A time course analysis of CSF-decorin in *App*^*NL-F/NL-F*^ mice revealed an increase of CSF-decorin levels at three months of age even before the establishment of Aβ plaque deposition followed by a continuous decrease upon Aβ pathology development, which is similar to the CSF-decorin alterations in a + t − human subjects. An increase in CSF-decorin was also found in the *App*^*NL-G-F/NL-G-F*^ mice by ELISA. The discrepancy between MS and decorin ELISA data of the 12–13 months old *App*^*NL-G-F/NL-G-F*^ mice may be due to differences in detection of different proteolytic fragments of decorin by the two methods (decorin was identified and quantified based on five to 11 different peptides, one of which contains a glycosylation site, in the MS-based proteomic approach, peptide list for decorin in Additional file 2: Table S2). Additionally, decorin is a proteoglycan which has several glycosylation sites, and the glycosylation levels may differ between the two *App* knock-in models. It is likely that the glycosylated and non-glycosylated peptides of decorin may not all be identified in the MS analysis since they have different mass-to-charge ratio.

Decorin has previously been shown to activate autophagy in endothelial cells, through VEGFR2/AMPKα activation and mTOR inhibition [39, 40], and in breast carcinoma cells [41] and activates autophagy flux in glioma cells [42] and intestinal cells [43]. We here extend this to include also neuronal autophagy, where autophagy-lysosomal pathway could be stimulated in vitro by decorin mainly via enhancing lysosomal degradation. Such activation may have implications for Aβ metabolism since Aβ is degraded through the autophagy-lysosomal system. Additionally, this autophagic effect by decorin may also involve JNK/MAPK signaling pathways which is involved in autophagy-mediated cell death [44]. This is interesting since autophagy is altered in AD [45, 46] as well as in the AD mouse models [47, 48]. Since impaired mitophagy contributes to the early AD related pathology [49] which can be ameliorated by mitophagy inducers [50] and decorin could activate mitophagy in breast carcinoma cells [41, 51], it will be interesting to analyze mitophagy alterations by decorin in the neurons which may give us a clue if decorin can be a potential therapeutic target for the early AD.

In summary, CSF-decorin levels are increased both in *App* knock-in and APP23 mice, especially in *App*^*NL-F/NL-F*^ mice and in a + t − preclinical AD subjects during early Aβ amyloidosis. Importantly, in these subjects, the CSF-decorin levels correlate with the standard CSF-Aβ42, CSF-t-tau and CSF-p-tau biomarkers and could predict a subclass of AD patients with potential ChP alterations. Therefore, changes in CSF-decorin, being an ECM protein, could reflect AD brain pathological changes driven by early Aβ amyloidosis and potentially in ChP. This opens up the possibility of decorin as a biomarker for these early changes.

## Conclusions

We have here identified the ECM protein decorin as similarly increased in mouse models exhibiting Aβ amyloidosis and in preclinical AD subjects. Furthermore, we show that CSF-decorin levels correlated with the core AD biomarkers CSF-Aβ42, CSF-t-tau and CSF-p-tau. Importantly, ROC analysis revealed that CSF-decorin could predict a subtype of AD subjects with potential ChP alterations. Lastly, human Aβ42 increases decorin secretion from neurons which activates autophagy-lysosomal pathway via enhancement of lysosomal degradation.

## Supplementary Information

**Additional file 1: Table S1.** Phosphorylation profiling of decorin treated and non-treated mouse primary neurons.

**Additional file 2: Table S2.** Proteins identified from label-free MS of mouse CSF.

**Additional file 3: Table S3.** Significantly altered ECM associated proteins in mouse CSF identified by MS and PEA.

**Additional file 4: Fig. S1.** Comparison of the CSF proteomes of the two *App* knock-in models reveals alterations in ECM proteins. **a** Volcano plots displaying the changes in protein levels in CSF from *App*^*NL-F/NL-F*^ vs *App*^*NL-G-F/NL-G-F*^ mice. Dash line: *p* = 0.05. **b** Heatmap of significantly (*p* < 0.05) altered proteins in *App*^*NL-F/NL-F*^ mice as compared to *App*^*NL-G-F/NL-G-F*^ mice. Proteins were sorted into upregulated and downregulated proteins in the *App*^*NL-G-F/NL-G-F*^ mice and by significance from highest to lowest (top to bottom).

**Additional file 5: Table S4.** Significantly altered ECM associated proteins in mouse CSF determined by PEA.

**Additional file 6: Table S5.** Description of the participants in EMIF-AD MBD cohort.

**Additional file 7: Table S6.** Proteome comparison of *App*^*NL-F/NL-F*^ and *App*^*NL-G-F/NL-G-F*^ mice vs NC/MCI/AD (abnormal-amyloid/abnormal-tau subjects).

**Additional file 8: Table S7.** Proteome comparison of *App*^*NL-F/NL-F*^ and *App*^*NL-G-F/NL-G-F*^ mice vs NC/MCI/AD (abnormal-amyloid/normal-tau subjects).

**Additional file 9: Fig. S2.** CSF-decorin negatively correlates with CSF-t-tau and CSF-p-tau in the whole EMIF-AD MBD cohort. **a** Correlations of CSF-DCN and CSF-Aβ42, **b** CSF-DCN and CSF-t-tau, **c** CSF-DCN and CSF-p-tau in the whole EMIF-AD MBD cohort (n = 310), including NC (n = 139), MCI (n = 92) and AD (n = 79) were analyzed. The regression coefficients (Beta) and *p*-values are displayed. *DCN* decorin.

**Additional file 10: Fig. S3.** Decorin is expressed in vessels on the brain surface but not in vessels of brain parenchyma. **a** Double immunostaining of decorin with αSMA (vascular smooth muscle cell marker) in 12 months old *App*^*wt/wt*^ mouse brain showing decorin expression in the arteries and **b** veins of the brain surface, but not **c** in the vessels of brain parenchyma. Scale bars, 200 µm (**a**, **b**), 100 µm (**c**). **d** Immunostaining of decorin in 12 months old *App*^*wt/wt*^, *App*^*NL-F/NL-F*^ and *App*^*NL-G-F/NL-G-F*^ mouse brains showing decorin expression in the vessels under the hypothalamus. Scale bars, 200 µm. **e** The intensities were quantified. (n = 4). Data in (**e**) were analyzed by one-way ANOVA followed by Dunnett’s multiple comparisons test. Data are represented as mean ± SEM. *DCN* decorin, *a* artery, *v* vein, *ns* not significant.

**Additional file 11: Fig. S4.** CSF-decorin levels correlates with Aβ pathology in the brains of *App*^*NL-F/NL-F*^ mice. **a** Spearman’s correlation analysis of parameters related to Aβ plaque load and decorin levels between 12 months old *App*^*NL-F/NL-F*^ and *App*^*wt/wt*^ mice, **b** *App*^*NL-G-F/NL-G-F*^ and *App*^*wt/wt*^ mice. The corresponding figures for each parameter are denoted. The correlation coefficients are displayed (*p < 0.05, **p < 0.01, ***p < 0.001). The red color represents positive correlation, and the green color represents negative correlation according to the scale bars in the right column.

**Additional file 12: Fig. S5.** Autophagy main upstream pathways are not affected by decorin in the neurons. Phosphorylation profiling of proteins involved in autophagy upstream pathways in non-treated and decorin-treated mouse primary neurons.

## Data Availability

The EMIF-AD mass spectrometry proteomics data have been deposited to the ProteomeXchange Consortium via the PRIDE partner repository with the dataset identifier 10.6019/PXD019910. Mouse CSF proteomic data will be uploaded to PRIDE database.

## Abbreviations

CSF: Cerebrospinal fluid
AD: Alzheimer’s disease
Aβ: Amyloid-β
MS: Mass spectrometry
ECM: Extracellular matrix
ChP: Choroid plexus
BBB: Blood–brain-barrier
BCSFB: Blood-cerebrospinal fluid barrier
FAD: Familial AD
PCA: Principle component analysis
PEA: Proximity extension assay
DCN: Decorin
ROC: Receiver operating characteristic
AUC: Area under the curve
PV: Parvalbumin
SRIF: Somatotropin release-inhibiting factor
Tg: Transgenic
ELISA: Enzyme-linked immunosorbent assay
NC: Normal cognition
MCI: Mild cognitive impairment
LC3: Microtubule associated protein 1 light chain 3
LAMP1: Lysosomal-associated membrane protein 1
TFEB: Transcription factor EB
