# Supplementary material for: Increased CSF-decorin predicts brain pathological changes driven by Alzheimer’s Aβ amyloidosis
Source: Acta Neuropathol Commun. 2022 Jul 4;10:96. doi: 10.1186/s40478-022-01398-5 (PMC9254429; doi:10.1186/s40478-022-01398-5)
Supplement: Supplementary file 3 — Additional file 3: Table S3. Significantly altered ECM associated proteins in mouse CSF identified by MS and PEA. [file 40478_2022_1398_MOESM3_ESM.docx]

**Table S3: Significantly altered ECM-associated proteins in mouse CSF identified by MS and PEA.**

| **ECM protein** | **Method** | **Alterations (log2 fold)** | ***p*-value** |
| --- | --- | --- | --- |
| Decorin | MS | 1.05* | 0.003 |
|  |  | 1.05^‡^ | 0.013 |
| Lumican | MS | 1.03* | 0.01 |
|  |  | 1.04^‡^ | 0.002 |
| Collagen alpha-1(I) chain | MS | 0.95^#^ | 0.004 |
|  |  | 1.06^‡^ | 0.002 |
| Basement membrane-specific heparan sulfate proteoglycan core protein | MS | 0.97^#^ | 0.01 |
| Fibronectin | MS | 0.98* | 0.049 |
|  |  | 0.97^#^ | 0.038 |
| SPARC-like protein 1 | MS | 0.97* | 0.022 |
|  |  | 0.97^#^ | 0.04 |
| Fibulin-1 | MS | 0.96^#^ | 0.041 |
| Vitronectin | MS | 0.97^#^ | 0.042 |
| Ecm1 protein | MS | 0.97^#^ | 0.045 |
| Procollagen C-endopeptidase enhancer protein | MS | 1.02^‡^ | 0.033 |
| CCN family member 4 | PEA | 1.51^‡^ | 0.004 |
| Matrilin-2 | PEA | 0.93* | 0.016 |
|  |  | 0.87^#^ | 0.008 |

**App^NL-F/NL-F^* vs *App^wt/wt^*; ^#^*App^NL-G-F/NL-G-F^* vs *App^wt/wt^*; ^‡^*App^NL-F/NL-F^* vs *App^NL-G-F/NL-G-F^*
