# Supplementary figures and images for: Increased CSF-decorin predicts brain pathological changes driven by Alzheimer’s Aβ amyloidosis
Source: Acta Neuropathol Commun. 2022 Jul 4;10:96. doi: 10.1186/s40478-022-01398-5 (PMC9254429; doi:10.1186/s40478-022-01398-5)

Fig. S1

a

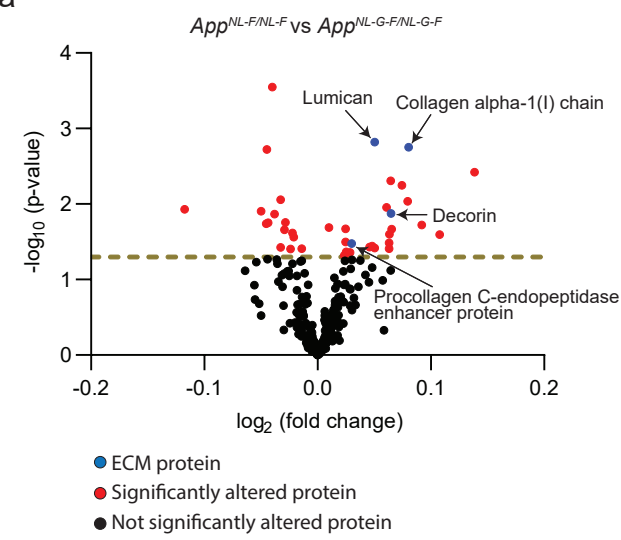

b

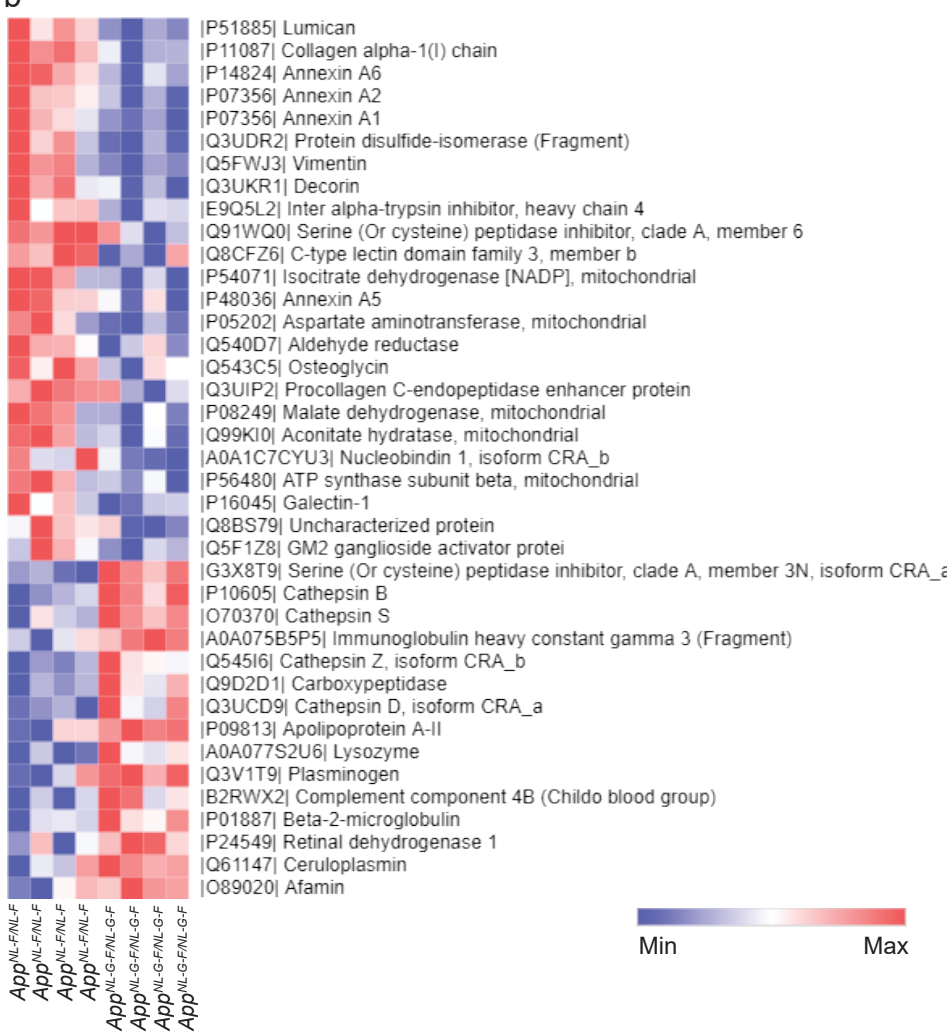

Supplement: Supplementary file 4 — Additional file 4: Fig. S1. Comparison of the CSF proteomes of the two App knock-in models reveals alterations in ECM proteins. a Volcano plots displaying the changes in protein levels in CSF from AppNL-F/NL-F vs AppNL-G-F/NL-G-F mice. Dash line: p = 0.05. b Heatmap of significantly (p < 0.05) altered proteins in AppNL-F/NL-F mice as compared to AppNL-G-F/NL-G-F mice. Proteins were sorted into upregulated and downregulated proteins in the AppNL-G-F/NL-G-F mice and by significance from highest to lowest (top to bottom). [file 40478_2022_1398_MOESM4_ESM.pdf]

Fig. S2

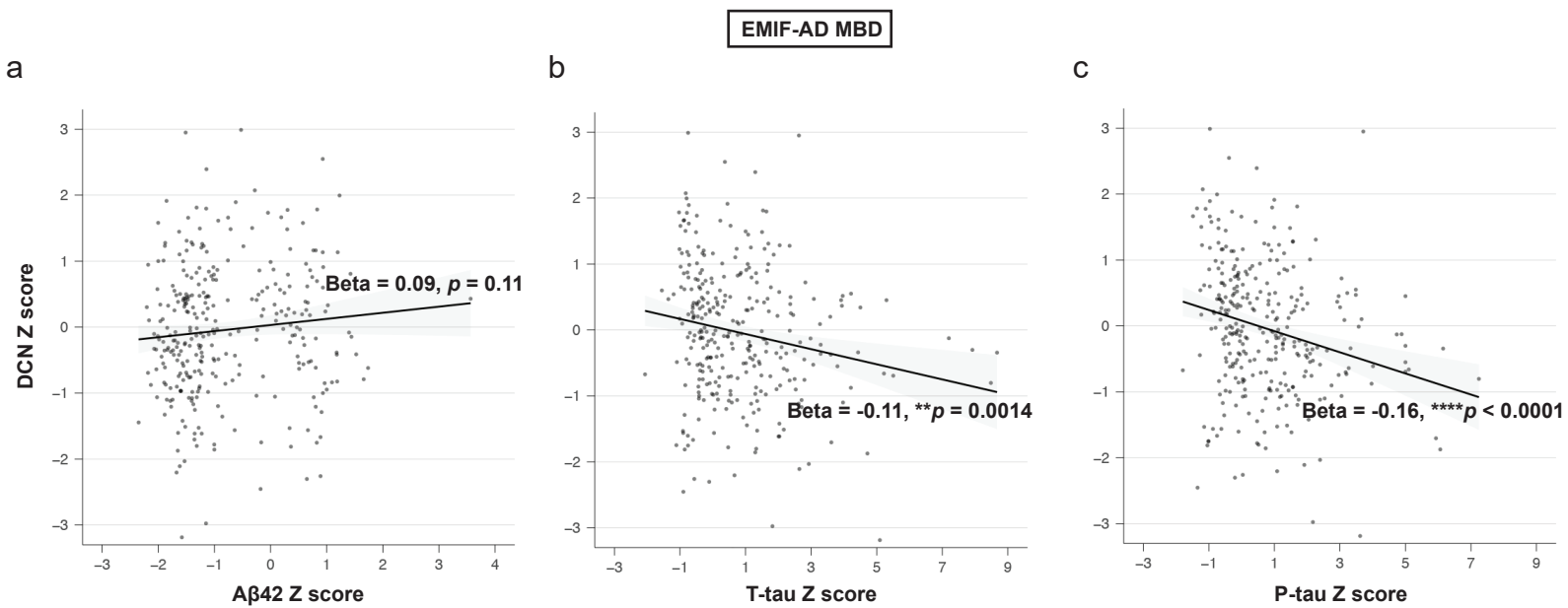

Supplement: Supplementary file 9 — Additional file 9: Fig. S2. CSF-decorin negatively correlates with CSF-t-tau and CSF-p-tau in the whole EMIF-AD MBD cohort. a Correlations of CSF-DCN and CSF-Aβ42, b CSF-DCN and CSF-t-tau, c CSF-DCN and CSF-p-tau in the whole EMIF-AD MBD cohort (n = 310), including NC (n = 139), MCI (n = 92) and AD (n = 79) were analyzed. The regression coefficients (Beta) and p-values are displayed. DCN decorin. [file 40478_2022_1398_MOESM9_ESM.pdf]

**App<sup>wt/wt</sup>**

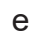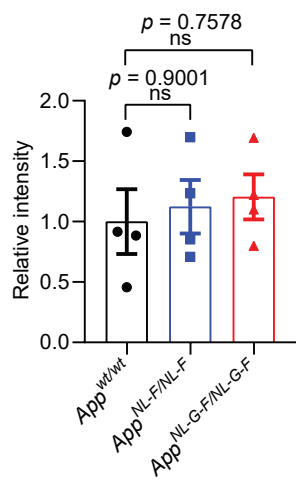

Supplement: Supplementary file 10 — Additional file 10: Fig. S3. Decorin is expressed in vessels on the brain surface but not in vessels of brain parenchyma. a Double immunostaining of decorin with αSMA (vascular smooth muscle cell marker) in 12 months old Appwt/wt mouse brain showing decorin expression in the arteries and b veins of the brain surface, but not c in the vessels of brain parenchyma. Scale bars, 200 µm (a, b), 100 µm (c). d Immunostaining of decorin in 12 months old Appwt/wt, AppNL-F/NL-F and AppNL-G-F/NL-G-F mouse brains showing decorin expression in the vessels under the hypothalamus. Scale bars, 200 µm. e The intensities were quantified. (n = 4). Data in (e) were analyzed by one-way ANOVA followed by Dunnett’s multiple comparisons test. Data are represented as mean ± SEM. DCN decorin, a artery, v vein, ns not significant. [file 40478_2022_1398_MOESM10_ESM.pdf]

Fig. S4

a

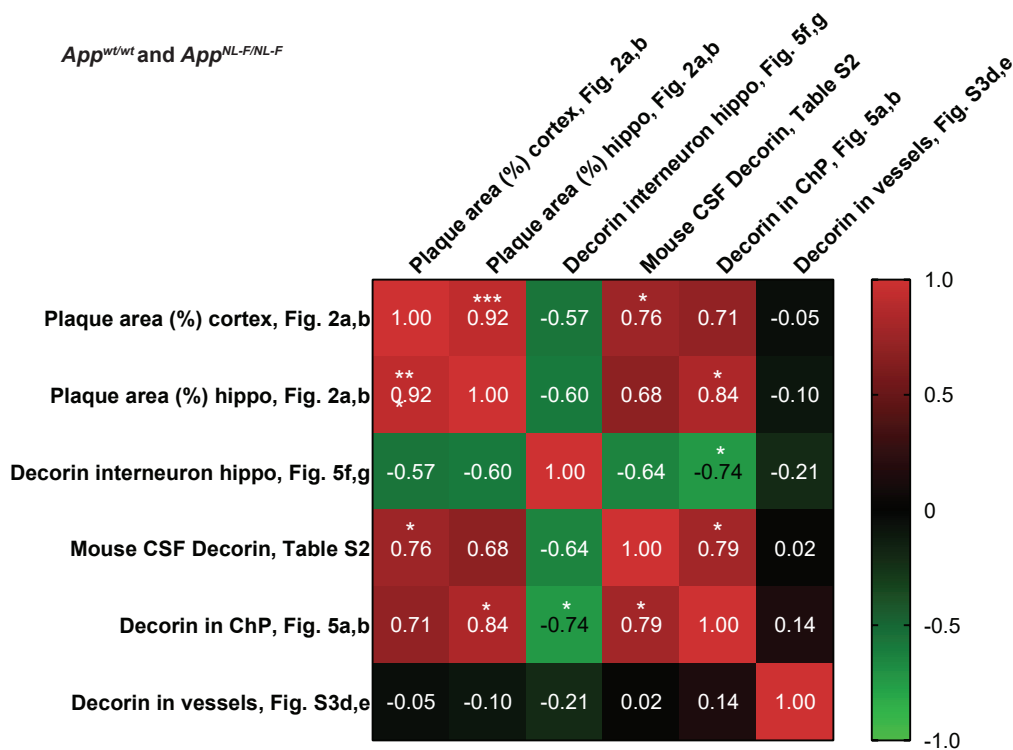

b

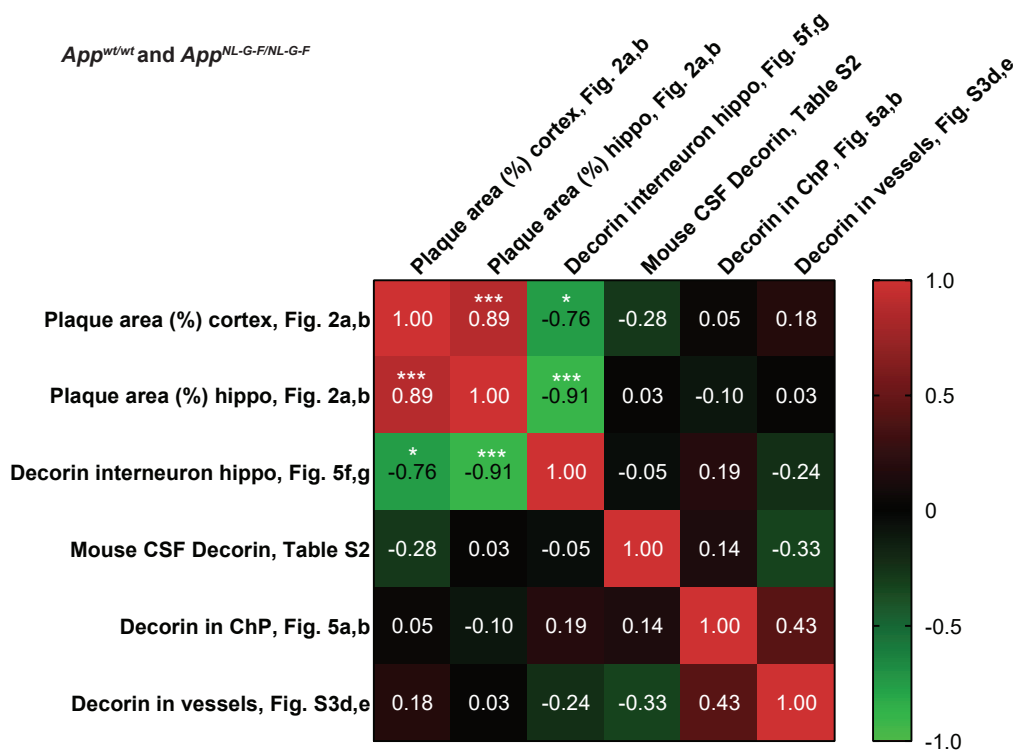

Supplement: Supplementary file 11 — Additional file 11: Fig. S4. CSF-decorin levels correlates with Aβ pathology in the brains of AppNL-F/NL-F mice. a Spearman’s correlation analysis of parameters related to Aβ plaque load and decorin levels between 12 months old AppNL-F/NL-F and Appwt/wt mice, b AppNL-G-F/NL-G-F and Appwt/wt mice. The corresponding figures for each parameter are denoted. The correlation coefficients are displayed (*p < 0.05, **p < 0.01, ***p < 0.001). The red color represents positive correlation, and the green color represents negative correlation according to the scale bars in the right column. [file 40478_2022_1398_MOESM11_ESM.pdf]

Fig. S5

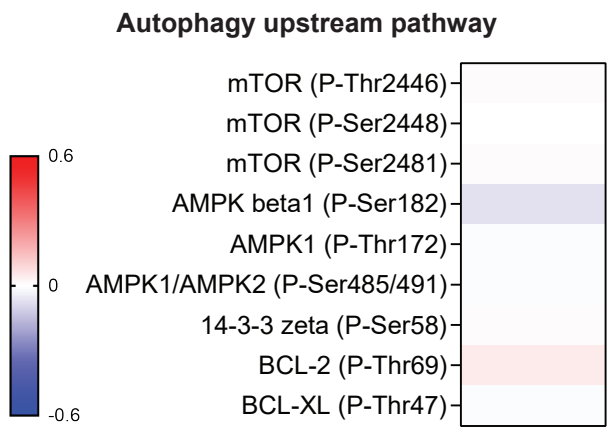

Supplement: Supplementary file 12 — Additional file 12: Fig. S5. Autophagy main upstream pathways are not affected by decorin in the neurons. Phosphorylation profiling of proteins involved in autophagy upstream pathways in non-treated and decorin-treated mouse primary neurons. [file 40478_2022_1398_MOESM12_ESM.pdf]
