# Supplementary material for: Increased CSF-decorin predicts brain pathological changes driven by Alzheimer’s Aβ amyloidosis
Source: Acta Neuropathol Commun. 2022 Jul 4;10:96. doi: 10.1186/s40478-022-01398-5 (PMC9254429; doi:10.1186/s40478-022-01398-5)
Supplement: Supplementary file 6 — Additional file 6: Table S5. Description of the participants in EMIF-AD MBD cohort. [file 40478_2022_1398_MOESM6_ESM.docx]

**Table S5: Description of the participants in EMIF-AD MBD cohort.**

|  | Healthy | | Alzheimer’s disease |
| --- | --- | --- | --- |
| N (subgroup n) | 82 | | 228 (NC 57, MCI 92, Dementia 79) |
| Age, mean of years (SD) | 61.1 (7) | | 68.1 (8) |
| Sex ratio, femeles/males | 47/35 | | 126/102 |
| Education, mean of years (SD) | 11.9 (3.5) | | 11.2 (3.5) |
| MMSE, mean of scores (SD) | 28.6 (1.3) | | 25.6 (3.9) |
| APOE e4, at least one allele | 14 | | 140 |
| Aβ_1-42_ pg/ml, mean (SD) | 0 (1) | | -2.8 (1.5) |
| t-tau pg/ml, mean (SD) | 0 (1) | | 4.4 (4.7) |
| Abnormal t-tau, n (%) | 0 (0) | | 151 (66) |
| p-tau pg/ml, mean (SD) | 0 (1) | | 2.1 (2.5) |
| Abnormal p-tau, n (%) | 7 (8.5) | | 149 (65) |
| Hippocampal volume, mean (SD) | | 0 (1) | -1.4 (1.5) |

NC: normal cognition, MCI: mild cognitive impairment, MMSE: mini-mental state examination.

More detailed information on the cohort as described previously [1, 2].

**REFERENCES**

1 Bos I, Vos S, Vandenberghe R, Scheltens P, Engelborghs S, Frisoni G, Molinuevo JL, Wallin A, Lleó A, Popp Jet al (2018) The EMIF-AD Multimodal Biomarker Discovery study: design, methods and cohort characteristics. Alzheimers Res Ther 10: 64 Doi 10.1186/s13195-018-0396-5

2 Tijms BM, Gobom J, Reus L, Jansen I, Hong S, Dobricic V, Kilpert F, ten Kate M, Barkhof F, Tsolaki Met al (2020) Pathophysiological subtypes of Alzheimer’s disease based on cerebrospinal fluid proteomics. Brain: Doi 10.1093/brain/awaa325
